# Patients’ Satisfaction with Lower-limb Prosthetic and Orthotic Devices and Service delivery in Sierra Leone and Malawi

**DOI:** 10.1186/s12913-017-2044-3

**Published:** 2017-02-01

**Authors:** Lina Magnusson, Gerd Ahlström

**Affiliations:** Department of Health Sciences, Faculty of Medicine, Lund University, P.O Box 187, SE 221 00 Lund, Sweden

**Keywords:** Assistive device, Disability, Low-income countries, Mobility, Orthosis, Prosthesis, Satisfaction, QUEST

## Abstract

**Background:**

People with disabilities have the right to personal mobility and available and affordable assistive technology, according to the Convention of Rights of Persons with Disabilities. The aims were to investigate similarities and differences between Sierra Leone and Malawi concerning participants’ mobility and satisfaction with their lower-limb prosthetic or orthotic device and related service delivery, and to identify variables associated with patients’ satisfaction with assistive devices and associated services in the entire study group from these two low-income countries.

**Methods:**

Questionnaires, including QUEST, were answered by 222 patients in Sierra Leone and Malawi.

**Results:**

Eighty-six per cent of assistive devices were in use, but half needed repair. One third of participants reported pain when using their assistive device. A higher percentage (66%) of participants in Sierra Leone had difficulties or could not walk at all on uneven ground compared with 42% in Malawi. The majority in both countries had difficulties or could not walk at all up and down hills, or on stairs. Participants in both countries were quite satisfied (mean 3.7–3.9 of 5) with their assistive device. Participants were most dissatisfied with: comfort (46%), dimensions (39%), and safety (38%) of their assistive device. In Sierra Leone participants were less satisfied than in Malawi with service delivery (mean 3.7; 4.4, *p* < .001). Access to repairs and servicing of their assistive device was considered the most important item. In Sierra Leone patients were less satisfied with follow-up services (41%) than patients in Malawi were (22%). The strongest association with satisfaction with assistive device was pain, and for satisfaction with service, country. The general condition of devices and the ability to walk on uneven ground were associated with both satisfaction with assistive devices and service received.

**Conclusions:**

Participants reported high levels of use and mobility with their assistive device, in spite of pain and difficulties walking on uneven ground, which were also associated with the level of satisfaction with the assistive device. Access to repairs and follow-up services were the most important to patients, and should be addressed. Country was associated with satisfaction with service, with participants in Sierra Leone significantly less satisfied.

**Electronic supplementary material:**

The online version of this article (doi:10.1186/s12913-017-2044-3) contains supplementary material, which is available to authorized users.

## Background

People with disabilities in low-income countries have the right to personal mobility and available and affordable assistive technology, such as prosthetic and orthotic services, according to the Convention of Rights of Persons with Disabilities (CRPD) [1, 2]. Furthermore, the WHO action plan for 2014–2021 [3] and the multiagency Global Cooperation on Assistive Technology scheme [4] have the objective of strengthening and extending rehabilitation services and assistive technology. Prosthetic and orthotic services are limited in low-income countries and need to be scaled up, as they have the potential to improve mobility and facilitate increased inclusion in society for amputees and persons with physical impairments. Factors important for patients’ satisfaction with prosthetic and orthotic devices used in low-income countries need to be identified in order to clarify which areas need prioritising in further, low-cost technology development [5] and improvements in service-delivery programmes.

Sierra Leone and Malawi are two low-income countries in sub-Saharan Africa and among the fifteen least-developed countries in the world, where the majority of the population lives under the ‘absolute poverty’ line [6]. Sierra Leone has a history of conflict and large violations of human rights, which occurred during the country’s civil war between 1991 and 2002 [7]; Malawi has a more stable history [6]. The population in Sierra Leone is 6.2 million people and in Malawi 16.8 million people [6]. Many low-income countries do not offer formal university education in the field of prosthetics/orthotics [3], which is the case in Sierra Leone and Malawi. This results in limited availability of prosthetic and orthotic services provided by qualified staff for persons with physical disabilities in low-income countries, although Tanzania offers an education programme where staff from English-speaking Africa are trained. At the time, in 2010/2011, the rehabilitation centres in Sierra Leone had four prosthetists/orthotists with university education and 15 prosthetic and orthotic technicians with on the job training employed [8]. In Malawi nine prosthetist/orthotists and five prosthetic and orthotic technicians were employed.

Appropriate technology for low-income countries needs to be affordable and must suit the environment of the user. Polypropylene technology, developed by the International Committee of the Red Cross (ICRC), is a commonly used low-cost technology in low-income countries [9, 10] and was used in Sierra Leone and Malawi for the production of prosthetic and orthotic devices together with traditional metal bar orthoses, most commonly for polio patients.

A literature review of lower-limb prosthetic technologies in developing countries indicated that there was a particular need for research related to policy, service delivery and patient outcomes [11]. Some product evaluation of assistive devices was performed, but further research and product development was also needed [11, 12]. A survey of a number of low-income countries indicated that the development of improved designs, using low-cost and durable components, needed to be addressed. Studies investigating patients’ capability, mobility and satisfaction with their assistive device, along with patients’ satisfaction with service delivery using low-cost technology in low-income countries, were very limited [13]. To our knowledge there are no previous studies investigating factors associated with patient mobility and satisfaction with low cost prosthetic and orthotic devices in low-income contexts.

## Aim

The aims were to investigate similarities and differences between Sierra Leone and Malawi concerning participants’ mobility and satisfaction with their lower-limb prosthetic or orthotic device and related service delivery, and to identify variables associated with participants’ satisfaction with assistive devices and associated services in the entire study group from these two low-income countries.

## Methods

This is a cross-sectional survey study in two low-income countries with correlative and comparative design.

### Sampling

Participants were identified and recruited from the local registers at the prosthetic and orthotic centre at all four rehabilitation centres in Sierra Leone [14] and from Lilongwe, Malawi [15]. The other rehabilitation centre in Malawi, the centre in Blantyre, which was not included in the sample, used the same technology as the included centre in Lilongwe. Staff at all centres had the same level of education. To be eligible to participate, participants needed to be aged fifteen or older, have a lower-limb disorder, and have received prosthetic and/or orthotic services between April 2009 and December 2010. A total of 749 patients fulfilled the inclusion criteria – 553 in Sierra Leone and 196 in Malawi.

In Sierra Leone, only a few telephone numbers were available from the patient registers, so local rehabilitation staff assisted in contacting patients through visits to homes, schools, workplaces, via community organisations, and through key people. One hundred and thirty nine of the 553 patients were located and subsequently asked to participate. In Malawi, contact details for 148 of the 196 patients were available from the centre’s register. Of those 148, a local staff member was able to contact 97 patients, who were asked to participate. Reasons for not participating were: illness (2), not being able to travel on public transport (3), and those said they would come but never did (9).

Dropout analysis in both countries revealed no statistically significant differences between the participants in the register who participated in the study (Sierra Leone *n* = 139 and Malawi *n* = 83) and those who did not (Sierra Leone *n* = 414 and Malawi *n* = 113), regarding sex, age, type of assistive device (prosthetic or orthotic), or level of assistive device (above-knee and below-knee). In Malawi, there was no significant difference regarding region of residence, but in Sierra Leone the relative number of participants representing Freetown, the capital city, was slightly lower than other regions.

### Participants’ demographics and characteristics

Participants included are presented in Table 1. The average age of participants was 35 years (range 15–81 years). The most common causes of impairments in Sierra Leone were violence and polio, while in Malawi, traffic accidents, non-healing wounds, and fractures were the most common causes. Of the total number of participants, 65% were prosthetic users and 35% orthotic users. The majority of prosthetic and orthotic devices were in use by participants for an average of nine hours a day, although in both countries about half of the assistive devices that were in use needed repairs. The majority of participants had no spare prosthetic or orthotic device. Use of wheelchairs or crutches rather than a prosthetic or orthotic device, or along with the device, was significantly more common in Sierra Leone than in Malawi.

**Table 1:** Demographics and characteristics in the study groups (*n* = 222)

	Entire study group *n* (%)	Sierra Leone *n* (%)	Malawi *n* (%)	*P*-value Chi Square *T-tests
Country of residence	222 (100)	139 (63)	83 (37)	
Age, *n* = 220				
Mean years, range	35 (15–81)	34 (15–81)	36 (16–74)	*.491
Sex, *n* = 222				
Female	75 (34)	39 (28)	36 (43)	.020
Male	147 (66)	100 (72)	47 (57)	
Rural/urban areas *n* = 222				
Living in cities	127 (57)	86 (62)	41 (49)	.069
Living in villages	95 (43)	53 (38)	42 (51)	
Level of income *n* = 222				
No income at all	85 (39)	49 (36)	36 (43)	.230
Irregular income	93 (42)	64 (47)	29 (35)	
Regular income from employment	42 (19)	24 (18)	18 (21)	
Ability to pay for costs associated with receiving the service appliances, accommodation, travel *n* = 222				
Yes	91 (41)	76 (55)	15 (18)	.000
No	131 (59)	63 (45)	68 (82)	
Type of assistive device n = 221				
Prosthesis	143 (65)	79 (57)	64 (78)	.001
Orthosis	78 (35)	60 (43)	18 (22)	
Level of assistive device *n* = 220				
Below-knee assistive devices	93 (42)	49 (35)	44 (53)	.009
*Below-knee prosthesis* *Below-knee orthosis*	83 (37) 10 (5)	47 (34) 2 (1)	36 (42) 8 (10)	
Above-knee assistive devices	129 (58)	90 (65)	39 (47)	
*Above-knee prosthesis* *Above-knee orthosis*	60 (27) 69 (31)	32 (23) 58 (42)	28 (34) 11 (13)	
General condition of device** *n* = 221				
Never used and Broken cannot be used	29 (13)	20 (15)	9 (10)	.730
In use but needs repair	105 (47)	64 (46)	41 (50)	
In use good condition	87 (39)	54 (39)	33 (40)	
Average of hours assistive device is used per day *n* = 211	9	9	9	*.721
Do you use crutches? n = 218				
Yes instead of device; Yes together with device	148 (68)	103 (76)	45(54)	
No	70 (32)	32 (24)	38 (46)	.001
Do you use a wheelchair? *n* = 220				
Yes instead of device; Yes together with device	33 (15)	28 (20)	5 (6)	.004
No	187 (85)	109 (80)	78 (94)	

** Assessment made by researcher LM

### Instruments and translation procedures

The questionnaires included questions evaluating patients’ mobility, the Quebec User Evaluation of Satisfaction with Assistive Technology questionnaire (QUEST), and specific questions related to satisfaction with prosthetic and orthotic devices and associated services (Additional file 1). Patients could also add their own comments to the items.

In order to evaluate users’ mobility with their assistive device, a number of specific questions were developed to reflect different gait situations. Six items were included for the ability to walk (rise from a chair; move around in the home; walk on uneven ground/roads; walk up and down a hill; walk on stairs) and two items related to the ability to use transport (get in and out of a car; get on and off a bus). The response alternatives supplied were: Yes, without any difficulty; Yes, with difficulty; No, not at all; Not applicable (Additional file 1).

QUEST is a standardised form comprising 12 items that identifies the user’s satisfaction and dissatisfaction in relation to assistive technology and service [16–18]. Eight relate to user satisfaction with their assistive devices (dimensions; weight; ease of adjustment; safety; durability; simplicity of use; comfort; effectiveness), while four relate to service delivery (service-delivery programme; repairs and servicing; quality of professional services; follow-up services) [16]. QUEST uses a five-level response scale: 1) Not satisfied at all; 2) Not very satisfied; 3) More or less satisfied; 4) Quite satisfied; and 5) Very satisfied [16]. QUEST has been demonstrated to be a valid and reliable assessment tool [17, 18].

Specific questions related to prosthetic and orthotic rehabilitation services were generated from a literature review of relevant questionnaires, checklists, and clinical experience [19, 20]. Questions about how often assistive devices caused pain and wounds/skin irritations were included. Additional questions were also asked about satisfaction with the assistive device and service: 1) The training received by the user to facilitate their usage of their assistive device; 2) The level of coordination of prosthetic and orthotic services with other rehabilitation professionals; 3) The look/appearance of the assistive device; 4) How easy it is to keep the assistive device clean; 5) The user’s ability to pay for costs associated with receiving the assistive device and service; 6) Whether there were opportunities for the user to express their views about the assistive device to the prosthetist/orthotist; 7) Trust and confidence in the prosthetist’s/orthotist’s capability of delivering a quality service.

Permission was received from the Institute of Matching Person and Technology to translate the English version of QUEST 2.0 into Krio, which is spoken in Sierra Leone, and into Chichewa, spoken in Malawi. For the translation procedures, the following steps were taken: forward translation, expert panel back-translation, pre-testing, and cognitive interviewing [21]. The forward translation of the questionnaire from English to the local language was conducted by three different translators in Sierra Leone and in Malawi. In Sierra Leone, the translators were teachers working at the Sierra Leonean language department of the Freetown Teachers College. In Malawi, the translators had a background working in rehabilitation. Internal consistency/homogeneity for the English, Krio and Chichewa versions of the QUEST sub-scales ‘satisfaction with assistive device’ and ‘satisfaction with service’ were good, with Cronbach’s alpha values above 0.70 [22] except for the ‘satisfaction with service’ sub-scale in the Chichewa translation, where the Cronbach’s alpha value was 0.43.

### Data collection

Data collection was conducted between November 2010 and February 2011. Elapsed time since receiving prosthetic and/or orthotic services and the survey varied between 1 week and up to 1 year and ten month. Questionnaires were read to all participants because of low literacy levels. In Sierra Leone, they were read in English to 62 participants, in Krio to 77, and/or interpreted partly to another tribal language – Kono, Themne, or Limba. In Malawi, questions were read in English to 28 participants and in Chichewa to 55 participants. Questions were read to the participants in English by LM or in local languages by a trained research assistant. During data collection triangular seating [23] was used in cases when an interpreter was required. In Sierra Leone, data were collected in participants’ homes or villages (*n* = 32), in schools, workplaces, training centres (*n* = 31), sports grounds (*n* = 5), or at the rehabilitation centre (*n* = 71). In Malawi, data were collected at the rehabilitation centre (*n* = 83).

For all patients, the general condition of assistive devices was evaluated by LM, a Swedish certified prosthetist/orthotist. The general condition of assistive devices was classified as one of: Never used; Broken, cannot be used; In use but needs repair; In use, good condition.

### Data analysis

The QUEST 2.0 manual [16] was followed when summarising QUEST total scores for satisfaction with assistive device and service. In order to create large-enough groups to facilitate a statistical analysis, the two responses ‘Never used’ and ‘Broken, cannot be used’ for describing a device’s condition were combined. In addition, the four response options ‘Always’ and ‘Often’, along with ‘Seldom’ and ‘Never’, which were used for questions related to whether the assistive device caused pain and wounds/skin irritations, were combined into two options. Furthermore, the options ‘Yes, with difficulty’ and ‘No, not at all’ were combined due to small numbers when comparing mobility.

In order to identify differences between participants in Malawi and Sierra Leone in relation to demographics, participants characteristics, condition of assistive devices, mobility, and satisfaction with assistive devices and service delivery, chi-square tests, Mann-Whitney U tests (two-sided), or t-tests (two-sided) were used, dependent of the scale level of the data. P-values < 0.05 were considered to be statistically significant. SPSS 19 was used for statistical analyses.

The qualitative comments were analysed by manifest content analysis [24].

Data from both countries were combined in order to get acceptable statistical power. Linear regression analysis was conducted to explore which variables were associated with satisfaction with assistive device and services using the combined dataset. Simple linear regression analyses of 23 variables were initially conducted separately for the two outcomes of sub-scale scores for satisfaction with assistive device and service. Variables with p-values of less than 0.1 were included in a multiple regression analysis with backward elimination and in final multiple regression analysis. Both variables relating to general condition of device were included in the final model, even though only one of them proved to be significant. Residual analysis was conducted on the final models, and showed linear regression analysis was appropriate for use.

## Results

### Similarities and differences between Sierra Leone and Malawi

#### Participants’ mobility with their lower-limb prosthetic or orthotic device

Use of assistive devices was found to improve the ability of participants in both Sierra Leone and Malawi to walk. The majority of participants could walk more than one kilometre when using their prosthetic or orthotic device [101 (73%) in Sierra Leone and 49 (59%) in Malawi]; however, only a third could manage this distance without using their prosthesis or orthosis. In Sierra Leone, 76 (55%) participants could manage to move 100 metres or more without their prosthesis or orthosis, in comparison to 17 (21%) in Malawi (*p* <0.001). In Sierra Leone 12 participants (9%) reported that they could not walk at all, or could walk a few metres, with their assistive device, in comparison to 20 participants (24%) in Malawi (*p* = 0.002). Without their assistive device a higher percentage of participants were at not able to walk at all [30 (22%) in Sierra Leone and 40 (48%) in Malawi].

The majority of participants had the ability to rise from a chair [97 (71%) in Sierra Leone and 64 (77%) in Malawi] and move around in their home [108 (79%) in Sierra Leone and 65 (81%) in Malawi] without difficulties. More than half of the participants experienced difficulties or could not walk at all on uneven ground [90 (66%) in Sierra Leone and 34 (42%) in Malawi], up and down hills [102 (77%) in Sierra Leone and 62 (78%) in Malawi], and on stairs [90 (67%) in Sierra Leone and 47 (61%) in Malawi]. A higher percentage of participants in Sierra Leone could not or had difficulties walking on uneven ground, compared to participants in Malawi (*p* <0.001). About half of participants were able to travel by car [70 (53%) in Sierra Leone and 45 (57%) in Malawi] or by bus [62 (48%) in Sierra Leone and 44 (56%) in Malawi] without difficulty. Furthermore, about half of the participants reported that they did not have, or sometimes did not have, the opportunity to access prosthetic and orthotic workshops or rehabilitation services due to distance, cost, availability of transport, or lack of personal assistance [55 (40%) in Sierra Leone and 59 (71%) in Malawi].

#### Participants’ satisfaction with their lower-limb prosthetic or orthotic device and related service delivery

Approximately one third of participants ‘always or often’ experienced pain related to use of their device [46 (34%) in Sierra Leone and 33 (40%) in Malawi], and only a few participants reported that they never experienced pain related to its use [12 (9%) in Sierra Leone and 6 (7%) in Malawi]. Wounds or skin irritations related to use of prosthetic or orthotic devices were experienced ‘always or often’ by 37 participants (27%) in Sierra Leone and 22 participants (27%) in Malawi. Only one quarter of the participants ‘never’ experienced wounds or skin irritations [30 (22%) in Sierra Leone and 21 (25%) in Malawi].

The results of QUEST showed that participants were quite satisfied with their assistive device, and also quite or very satisfied with the services received. Participants in Sierra Leone were significantly less satisfied with service delivery than participants in Malawi (*p* < 0.001) – see Table 2.

**Table 2:** QUEST total scores of patients’ rating of satisfaction with assistive device and service

QUEST	Entire study group *n* = 222		Sierra Leone *n* = 139		Malawi *n* = 83	
Scale (1–5)	Mean (SD)	Median (IQR)	Mean (SD)	Median (IQR)	Mean (SD)	Median (IQR)
Satisfaction with assistive device, total score	3.8 (.7)	3.9 (1.1)	3.7 (.8)	3.8 (1.1)	3.9 (.7)	4.0 (1.0)
Satisfaction with services, total score	4.0 (1.0)	4.3 (1.5)	3.7* (1.0)	4.0 (1.8)	4.4* (.7)	4.7 (1.0)

Response scale: 1) Not satisfied at all; 2) Not very satisfied; 3) More or less satisfied; 4) Quite satisfied; 5) Very satisfied [16]

*T test *p* < 0.001, SD: standard deviation; IQR: Interquartile range

Table 3 shows that the items participants were most dissatisfied with were: comfort, dimensions and safety of assistive device, in both Sierra Leone and Malawi. In Sierra Leone a high proportion of participants were significantly less satisfied with follow-up services and repairs and services of assistive devices than participants in Malawi were.

**Table 3:** Participants’ rating of variables of satisfaction with assistive devices and services, using the QUEST instrument

		Entire study group			Sierra Leone			Malawi		
QUEST	*n*	% participants Not satisfied^a^	% participants Satisfied^b^	*n*	% participants Not satisfied^a^	% participants Satisfied^b^	*n*	% participants Not satisfied^a^	% participants Satisfied^b^	Chi-Square test *P*-value
Assistive device										
Dimension	221	39	61	139	37	63	82	40	60	.676
Weight	221	30	70	139	30	70	82	30	70	.876
Adjustment	221	26	74	139	25	75	82	29	71	.433
Safety	221	38	62	139	35	65	82	42	58	.357
Durability	215	28	72	137	30	70	78	24	76	.381
Easy to use	220	27	73	139	21	79	82	37	63	.012
Comfort	220	46	54	139	42	58	81	51	49	.240
Effectiveness	222	33	67	139	33	67	81	33	67	.971
Service										
Service delivery	220	23	77	138	30	70	82	11	89	.001
Repairs and services	209	27	73	132	39	61	77	8	92	.000
Professional Service	220	15	86	138	15	85	82	15	85	.977
Follow up	209	34	66	136	41	59	73	22	78	.005

^a^Not satisfied: scores 1, 2, 3 (not at all satisfied, not very satisfied more or less satisfied)

^b^Satisfied: scores 4, 5 (quite satisfied and very satisfied)

Complementary questions indicated that in both countries, participants reported high levels of satisfaction, on a scale 1–5, regarding training received (mean 4.4 in Sierra Leone and 4.4 Malawi), coordination between several professionals (mean 4.3 in Sierra Leone and 4.4 Malawi), and ease of keeping their assistive device clean (mean 4.4 in Sierra Leone and 4.4 Malawi). However, participants in Sierra Leone were significantly less satisfied with the cosmetic appearance of their device than participants in Malawi were (mean 3.5 in Sierra Leone and 4.4 in Malawi, *p* < 0.001).

A higher percentage of participants in Malawi reported that they did not have the ability to pay for costs associated with receiving prosthetic and orthotic services, including accommodation and travel to rehabilitation centres – 68 patients (82%), compared to 63 patients (45%) in Sierra Leone, *p* < 0.001. In Sierra Leone a higher percentage (80%) of participants indicated that staff gave them the opportunity to express their views about the device compared to 59% of participants in Malawi. The majority of participants in both countries trusted and had confidence that their prosthetist/orthotist was capable of delivering a quality service; see Table 4.

**Table 4:** Participants’ views about service delivery measured by study specific questions

	Entire study group *n* = 222 (%)	Sierra Leone *n* = 139 (%)	Malawi *n* = 83 (%)	*P*-value Chi Square
The prosthetist/orthotist or technician gives me the opportunity to express my views about my assistive device *n* = 216				
Completely true	156 (72)	108 (80)	48 (59)	
Sometimes true	35 (16)	20 (15)	15 (19)	
Completely false	25 (12)	7 (5)	18 (22)	<0.001
I trust and have confidence that my prosthetist/orthotist is capable of delivering a quality service *n* = 221				
Completely true	183 (83)	115 (83)	68 (82)	
Sometimes true	29 (13)	18 (13)	11 (13)	
Completely false	9 (4)	5 (4)	4 (5)	.907

Participants were asked to choose what they considered to be the three most important items among the 12 included in QUEST. The entire study group (*n* = 222) in Sierra Leone and Malawi reported that access to repairs and servicing of their assistive device was most important, followed by provision of follow-up services and durability of the device. The same items were considered the most important in Malawi (*n* = 83), while in Sierra Leone (*n* = 139), follow-up services, access to repairs and servicing and comfort of their assistive device were scored as the three most important items.

Manifest content analysis was performed on 1304 comments (886 comments in Sierra Leone and 418 comments in Malawi) related to problems experienced with assistive devices and service delivery. The three main concerns in Sierra Leone were related to lack of comfort and pain experienced when using the assistive device (148 comments by 78 (56%) participants); problems related specifically to service delivery, including the fact that the patients could not cover associated expenses (145 comments by 94 (68%) participants); and feelings that there were limitations to the effectiveness of their assistive device (107 comments by 69 (50%) participants). The three main problems in Malawi were related to lack of comfort and pain when using the assistive device (99 comments by 62 (75%) participants); limitations in the effectiveness of the assistive device (55 comments by 45 (54%) participants); and problems related to poor dimensioning of the assistive device (41 comments by 40 (48%) participants).

### Variables associated with satisfaction with assistive devices and services

Table 5 show the result of simple linear regression analyses of 23 initial variables. The final multiple regression models are presented in Table 6. The variables which were significantly associated (*p* < 0.1) with satisfaction with assistive device were: pain, general condition of the device, ability to walk on uneven ground/roads, ability to walk on stairs, and ability to get in and out of a car (Adjusted R^2^ = 30%, F-ratio = 15). Pain, general condition of the device and ability to walk on uneven ground were the three strongest predictors. The variables which were significantly associated (*p* < 0.1) with satisfaction with service were: country, general condition of the device, ability to walk on uneven ground, ability to pay for costs associated with receiving the service of the device, accommodation, travel, and ability to walk on stairs (Adjusted R^2^ = 38%, F-ratio = 21). Country, general condition of the device and the ability to walk on uneven ground were the three strongest predictors.

**Table 5:** Variables included in regression analysis

	Total study group	
Independent variables included in simple regression analysis	Satisfaction with assistive device *p*-value	Satisfaction with service *p*-value
Background variables		
Country [Malawi; Sierra Leone]	.291	<.001*
Sex [Female; Male]	.477	.786
Age	.617	.644
Urban/ rural areas [Living in cities; Living in villages]	.642	.425
Level of income [No income at all; Regular income from employment]	.295	.994
[No income at all; Sometimes income]	.629	.456
Ability to pay for costs associated with receiving the services	<.001*	.058*
Type of assistive device [Prosthesis; Orthosis]	.132	<.001*
Level of assistive device [Below-knee assistive devices; Above-knee assistive devices]	.008*	<.001*
General condition of device [In use good conditoin; broken cannot be used] [In use but needs repair; Broken, cannot be used]	.001* .371*	<.001* .002*
Hours assistive device is used per day	<.001*	.005*
Use of crutches [Yes, instead of device and Yes, together with device; No]	.001*	.001*
Use of wheelchair [Yes, instead of device and Yes, together with device; No]	.404	.058*
Mobility variables [Not at all, 0 metres and a few metres; About 100 metres and About a kilometre or more]		
Walking distance without assistive device	.589	.051*
Walking distance with assistive device	.002*	.633
Mobility variables [Yes, without any difficulty; Yes, with difficulty and No, not at all]		
Ability to rise from a chair	.001*	.058*
Ability to move around the home	<.001*	<.001*
Ability to walk on uneven ground/roads	<.001*	<.001*
Ability to walk up and down a hill	<.001*	.118
Ability to walk on stairs	<.001*	<.001*
Ability to get in and out of a car	<.001*	<.001*
Ability to and get on and off a bus	<.001*	<.001*
Pain variables [Always and Often; Seldom and Never]		
Assistive device causes pain	<.001*	.631
Assistive device causes wounds/skin irritations	<.001*	.155

*Variables showing a significant association with the dependent variables in simple regression analysis *p* < 0.10 were entered into the multiple regression analysis

**Table 6:** Variables associated with QUEST total score for satisfaction with assistive device and service

	Total study group		
Variables	B	95% CI	*P*-value
Model: Satisfaction with assistive device^a^			
Constant/Intercept	2.92	2.61 to 3.23	
Pain [Seldom Never (1); Always Often (0)]	.37	.20 to .54	<.001
General condition of device [In use good condition (1); Broken, cannot be used (0)] [In use but needs repair (1); Broken, cannot be used (0)]	.54 .15	.24 to .84 -.14 to .45	<.001 .308
Ability to walk on uneven ground/roads [Yes, without difficulty (1); Yes, with difficulty and No, not at all (0)]	.35	.18 to .52	<.001
Ability to walk on stairs [Yes, without any difficulty (1); Yes, with difficulty and No, not at all (0)]	.23	.03 to .42	.023
Ability to get in and out of a car [Yes, without difficulties (1); Yes, with difficulty and No, not at all (0)]	.20	.01 to .39	.038
Model: Satisfaction with service^b^			
Constant/Intercept	3.33	2.92 to 3.74	
Country [Malawi (1); Sierra Leone (0)]	.63	.38 to .88	<.001
General condition of device [In use good condition (1); Never used and Broken, cannot be used (0)] [In use but needs repair (1); Never used and Broken, cannot be used (0)]	.86 .35	.46 to 1.26 -.04 to .74	<.001 .077
Ability to walk on uneven ground/roads [Yes, without difficulty (1); Yes, with difficulty and No, not at all (0)]	.56	.36 to .79	<.001
Ability to pay for costs associated with receiving the service appliances, accommodation and travel [Yes (1); No (0)]	.40	.17 to .64	.001
Ability to walk on stairs [Yes, without any difficulty (1); Yes, with difficulty and No, not at all (0)]	.34	.11 to .37	.004

^a^Satisfaction with assistive device: Adjusted R^2^ = 30%, F-ratio = 15

^b^Satisfaction with service: Adjusted R^2^ = 38%, F-ratio = 21

## Discussion

The main findings show that the majority of assistive devices were in use, but half needed repair. Patients were most dissatisfied with the comfort and dimensions of their assistive device. They reported pain related to use of assistive devices as a major problem and pain was the variable most strongly associated with satisfaction with assistive device; this needs to be addressed. Participants had difficulties or could not walk at all on uneven ground, walk up and down hills, and on stairs; improvements to low-cost technology need to be made. The strongest association with satisfaction with service was country. In Sierra Leone, participants were less satisfied than in Malawi with service delivery and follow-up services and had less ability to walk on uneven ground.

When investigating the usage of prosthetic and orthotic devices, about 90% of the prosthetic and orthotic devices were in use by participants. Our study showed similar results to those of preceding studies, and confirmed that the majority of the low-cost technology prosthetic and orthotic devices delivered were in use. In Vietnam, Cambodia and El Salvador, 93–100% of below-knee prostheses were in use [9, 25]. The average daily use of prostheses and orthoses was nine hours in both Sierra Leone and Malawi, which is consistent with previous studies from Vietnam, Cambodia, and El Salvador, where 8–15 hours of wear time per day was reported [9, 25–27]. Results indicate that while the low-cost technology prostheses and orthoses delivered to participants were used, 41% of participants in Sierra Leone and 10% of participants in Malawi preferred to use crutches rather than a prosthetic or orthotic device, at least at certain times. This indicated that the devices were not designed for high activity levels; in Sierra Leone, for example, it was common for amputees to play football and they would use only crutches. In Sierra Leone and Malawi, about 10% of participants had a spare device. An extra assistive device would make the participants less vulnerable if their regular assistive device broke. If resources are limited, the first priority should, however, be to provide prostheses and orthoses to those who have not yet received the service, rather than to provide participants with spare devices.

The majority of participants in Sierra Leone and Malawi could walk around their home and on level surfaces. The majority could also walk more than a kilometre; 73% in Sierra Leone and 59% in Malawi. In a Vietnamese study, 79% reported being active or having a high ambulation capacity [28]. Other studies reported that 100% of patients in Vietnam, 92% of patients in Cambodia, and 66% in El Salvador could walk more than a kilometre [9]. This difference in results could partly be due to the fact that in Sierra Leone and Malawi, a higher number of participants are using above-knee prosthetic and orthotic devices. It may also indicate that the prosthetic and orthotic devices and related services delivered in Sierra Leone, Malawi and El Salvador were of lower quality than those in Vietnam and Cambodia.

The ability to walk on uneven ground was associated with the level of satisfaction with both assistive devices and services. About half of the participants had difficulty or could not manage at all when walking on uneven ground, stairs, and slopes. This corresponds well with the results of the qualitative comments, where limitations to the effectiveness of the assistive device was the second largest category in Malawi and the third largest in Sierra Leone. Most previous studies have not investigated ambulation on challenging surfaces, but one study from Vietnam reported that 93% of prosthetic users could walk up and down steps [26]. A small study in India indicated that the design of orthotic devices had an impact on patient mobility and satisfaction [29]. The ability to walk on uneven ground and slopes was essential in both rural and urban areas, as the walking surfaces were unpaved and the rainy season created rough surfaces.

Polypropylene technology developed by the ICRC was used to produce assistive devices in both countries. Service providers should consider the design of the device so as to facilitate mobility for patients on challenging surfaces, and walking on uneven and sloped terrain [30–32]. They should also consider performing a dynamic alignment on challenging surfaces [33] as poor alignment of prosthetic and orthotic devices had been previously reported [5]. Furthermore, they should focus on the training of mobility skills with the prosthesis or orthosis [1]. ICRC polypropylene technology often results in prosthetic and orthotic devices with rigid ankles, which may explain some of the difficulties observed in this study regarding walking on slopes and stairs.

Gait training is often conducted by the prosthetist/orthotist if no physiotherapist is available. Increased gait training on challenging surfaces and in natural environments, along with providing patients with coping strategies, has been shown to improve gait in amputees using advanced technology prostheses [34], and potentially has the capacity to increase mobility for patients using low-cost technology prostheses and orthoses as well. Participants in Malawi had less difficulty walking on uneven ground than participants in Sierra Leone did; this could be an effect of the staff in Malawi being more qualified for the job than those in Sierra Leone.

Satisfaction with assistive devices was strongly associated with pain when using the assistive device; this also corresponds well with qualitative comments, where pain and lack of comfort was the largest category. Pain was considered to be an issue when walking longer distances. In addition, participants were most dissatisfied with the comfort and dimensions of their assistive device. Assistive devices with unsuitable dimensions will create discomfort and pain. In Vietnam, studies reported that between 2% and 10% of amputees experienced pain when using their ICRC polypropylene prosthesis [25, 27]. In addition, 3% of patients in a Cambodian study, and 28% in El Salvador, reported pain while using their prosthesis [9]. This study indicates a higher percentage of pain (34%–40%) related to usage of a prosthesis or orthosis than found in earlier studies of the same ICRC technology [9, 25, 27]. The reasons for this may be that in Sierra Leone and Malawi about half of the assistive devices needed repairs or replacing entirely, compared with the Vietnamese study; there, one quarter of the patients’ devices required repair work, and only 7% needed a new device [27]. A contributing factor to this difference might also be that the Vietnamese patients participated in the study roughly two months after receiving their prostheses [27], while in Sierra Leone and Malawi the time since receiving the service was longer.

In general, participants reported being quite or very satisfied with their assistive device and with the services received. Three studies from Vietnam including only one general question about satisfaction with lower-limb prosthetics show similar findings [25–27]. Ten per cent or fewer of patients were dissatisfied with their ICRC polypropylene prosthesis, while one third reported that amputees had limited ability to perform rigorous physical activity, but the majority were relatively satisfied with their prosthesis [25–27] and the service [26]. In Haiti, almost all patients with amputations expressed gratitude that prosthetic services were offered to them for free [35].

Participants in Sierra Leone were significantly less satisfied with service delivery than in Malawi. This was also confirmed by the results of regression analysis, which indicated that country was a variable associated with satisfaction with service delivery. A reason could be that the level of education of staff was lower in Sierra Leon than in Malawi. These services are best provided by clinicians who have received training within the field of prosthetics and orthotics and methods of using the ICRC technology [36, 37].

Participants in Malawi indicated that staff gave them few opportunities to express their views about the device, in contrast to findings from Sierra Leone. Differences between these countries were not expected and are of interest for further investigation. A study of Haitian amputees reported similar results to our study, in that patients were generally satisfied with prosthetic and orthotic services. Studies conducted in Iran [38], the Netherlands [39, 40], and the United States [41–43] indicate equal or less satisfaction compared to participants in Sierra Leone and Malawi. These studies did not, however, use the same instrument as this study to measure satisfaction. However, in open comments, participants in both Sierra Leone and Malawi reported numerous problems with their devices. Participants in Sierra Leone were less satisfied with the cosmetic appearance of their device than patients in Malawi were. Regarding the cosmesis of their prosthesis, patients in Haiti [35] and Iran [38] were even less satisfied.

The general condition of the device was associated with the level of satisfaction with assistive device and service. About half of the devices in use by participants needed repairs in Sierra Leone and Malawi, indicating that the current follow-up system, where participants were required to come back to the facility using their own means, was insufficient. Participants in both countries reported that access to repairs and servicing, provision of follow-up services and the durability of the assistive device were the most important issues. In addition, participants in Sierra Leone were less satisfied with follow-up services and repairs and services of assistive device than participants in Malawi were. These results were consistent with findings from Haiti, where persons with amputations who had received a prosthesis worried about the availability of long-term follow-up of assistive device and services [35]. These patients were given a three- or six-month follow-up appointment, and were also told that they could return any time before this six-month point if they had problems with their prosthesis. This was, however, poorly understood, as most participants who experienced pain or discomfort did not call for an earlier appointment [35]. Check-ups and regular maintenance of prosthetic and orthotic devices increases the lifespan of the device, and it is cheaper to undertake minor repairs than to entirely replace the prosthesis/orthosis [44]. In order to provide cost-effective programmes and deliver assistive devices and related services, funding needs to be allocated not only to new assistive devices, but also to follow-up services. It is likely that a lack of finances has a major effect on access to follow-up services and repairs.

Poverty affects access to prosthetic and orthotic services for persons with disabilities. The CRPD (Article 20) rules that states shall facilitate access to quality mobility aids and make them available at an affordable cost [1, 45]. Thirty nine per cent of the patients in this study had no income at all and only about one-fifth reported having a regular income. The majority of participants in Malawi and about half of the participants in Sierra Leone included in the studies struggled, or did not have the ability, to pay for transport and the costs associated with receiving services; these patients were entirely dependent on support from others. The results indicated that participants in both Sierra Leone and Malawi were limited in physically accessing services as described in the WHO joint position paper for mobility aids [45], due to long distances and limited access to transport. Fuel was very expensive [46] in relation to the level of income of the populations [6]. Previous studies from African countries have also confirmed that lack of transport and the inability of persons with disabilities living in rural areas to pay for transport reduced access to healthcare and rehabilitation [47]. Services were not affordable for all participants, as many lived in poverty. A study which investigated the inclusion of vulnerable groups in health policy in Africa indicated that loss of lower-limb mobility and expensive transportation affects access to healthcare [48]. About half of the participants in this study reported they could travel by car or bus without difficulties, although the majority of participants in Malawi managed to access the centre when funding for public transport was provided.

Patient satisfaction with assistive devices can be affected by expectations, previous experiences, life conditions, and healthcare values [49, 50]. It is likely that participants in Sierra Leone and Malawi had relatively low expectations. However, independent living aspects [51] are not included in the questionnaires and therefore it is important that reported satisfaction is not used as the sole indicator of quality. The results from Sierra Leone and Malawi suggested that participants value highly the prosthetic and orthotic device and services they receive. In Sierra Leone the prosthetic and orthotic devices were vital for people with physical disability to offer increased dignity [52].The fact that many of the participants had never received any prosthetic and orthotic services before the centre in Malawi, Lilongwe opened may have contributed to the high level of satisfaction reported. This study clearly indicates that including qualitative comments added a deeper understanding to the results. Participants was according to the results of the QUEST sub-scales quite satisfied with their assistive device, and quite or very satisfied with the services received. At the same time participants raised many problems in the qualitative comments included under each item included in QUEST. It seems that adding a qualitative component is preferable while conducting studies with the aim to identify where limited resources best should be spent in order to improve health services, especially in settings where participants are poor, have low expectations and can be potentially thankful for any health services they can get. In this study, one cannot rule out the possibility that participants felt that they needed to be thankful for the services they received and that this may have influenced the results.

In Sierra Leone and in Malawi, the most common cause of disability was trauma for participants using prosthetics and polio for participants using orthotics. Studies from Vietnam, Cambodia, and El Salvador show that 79% or more of patients had disabilities caused by trauma [9, 25–28]. These studies primarily included below-knee prosthetic users [9, 25, 26]. Earlier studies have included below-knee prosthetic users only and our study has in addition included above-knee prosthetic patients and orthotic patients. The comparisons therefore need to be considered with some caution. However, the average age of participants and the technology used in producing prostheses was similar.

## Conclusions

Participants reported high levels of use and mobility with their assistive device in both Sierra Leone and Malawi, but experienced high levels of pain and difficulties walking on uneven ground and on stairs. These aspects were also shown to be the most strongly associated with level of satisfaction with the assistive device. More than half of the assistive devices were in need of repair; this was also strongly associated with satisfaction with both assistive device and services, as well as with access to repairs and follow-up services, which were the most important to participants. There are no significant differences between Sierra Leone and Malawi in how satisfied participants were with their assistive devices. Country, together with general condition of the device, were the strongest variables associated with satisfaction with service, with participants in Malawi significantly more satisfied than participants in Sierra Leone.

The results revealed that the design and manufacture of prostheses and orthoses using low-cost technology needs be improved, specifically towards appropriate dimensions, increased comfort, and increasing the ability of patients to ambulate on challenging surfaces with their assistive device, as well as increasing patients’ ability to walk long distances with reduced pain. Increased or simulated ankle joint range of motion, careful dynamic alignment, more optimal dimensioning of assistive devices, and better training could facilitate the desired improvements. Access to repairs and follow-up services were important to participants, and should be addressed both by professionals operating within the rehabilitation field and policymakers, as it has the potential to improve mobility and satisfaction levels as well as reduce pain. In Sierra Leone, the quality of assistive devices and service delivery could be enhanced by addressing the education level of staff.

## Additional file

Additional file 1: Supplementary file the *Questionnaire Prosthetic and Orthotic Device and Services*. The questionnaire includes questions regarding the characteristics of participants and assistive devices, mobility, pain and satisfaction with assistive device. (DOCX 19 kb)

## Abbreviations

CRPD: Convention of Rights of Persons with Disabilities
ICRC: the International Committee of the Red Cross
QUEST: the Quebec User Evaluation of Satisfaction with Assistive Technology questionnaire

## References

[1] United Nations. The Convention on the Rights of Persons with Disabilities New York: United Nations; 2007. https://www.un.org/development/desa/disabilities/convention-on-the-rights-of-persons-with-disabilities.html. Accessed 12 Jan 2016.

[2] Borg J, Lindström A, Larsson S (2009). Assistive technology in developing countries: national and international responsibilities to implement the Convention on the Rights of Persons with Disabilities. Lancet.

[3] World Health Organization: Disability Draft WHO global disability action plan 2014–2021: Better health for all people with disability. In*.* Sixty-seventh World Health Assembly WHO 2014.

[4] World Health Organisation. Global Cooperation on Assistive Technology (GATE) 2016. http://www.who.int/phi/implementation/assistive_technology/phi_gate/en/ Accessed 13 Jan 2016.

[5] Wyss D, Lindsay S, Cleghorn WL, Andrysek J (2015). Priorities in lower limb prosthetic service delivery based on an international survey of prosthetists in low- and high-income countries. Prosthet Orthotic Int.

[6] United Nations Development Programme. Country profiles International Human Development Indicators 2015. www.hdr.undp.org/en/countries Accessed 24 Apr 2016.

[7] Assembly UNG (2011). National report submitted in accordance with paragraph 15 (a) of the annex to Human Rights Council resolution 5/1 Sierra Leone. In.

[8] Magnusson L, Ahlström G (2012). Experiences of Providing Prosthetic and Orthotic Services in Sierra Leone — the Local Staff’s Perspective. Disabil Rehabil.

[9] Jensen S, Raab W, Fisk J, Hartz C, Saldana A, Harte C (2006). Quality of polypropylene sockets for trans-tibial prostheses in low-income countries. Prosthet Orthot Int.

[10] Verhoeff T, Poetsma P, Glasser L, Tung H (1999). Evaluation of use and durability of polypropylene trans-ti bial prostheses. Prosthet Orthot Int..

[11] Borg J, Lindström A, Larsson S (2011). Assistive technology in developing countries: a review from the perspective of the Convention on the Rights of Persons with Disabilities. Prosthet Orthot Int.

[12] Andrysek J (2010). Lower-limb prosthetic technologies in the developing world: A review of literature from 1994–2010. Prosthet Orthot Int.

[13] Borg J (2011). Assistive technology, human rights and poverty in developing countries.

[14] Magnusson L, Ramstrand N, Fransson EI, Ahlström G (2014). Mobility and satisfaction with lower-limb prostheses and orthoses among users in Sierra Leone: a cross-sectional study. J Rehabil Med.

[15] Magnusson L, Ahlström G, Ramstrand N, Fransson EI (2013). Malawian Prosthetic and Orthotic Users’ Mobility and Satisfaction with their Lower – Limb Assistive Device. J Rehabil Med.

[16] Demers R, Weiss-Lambrou B, Bernadette S (2000). Quebec User Evaluation of Satisfaction with assistive Technology QUEST version 2.0 an outcome measure for assistive technology device.

[17] Wessels RD, De Witte LP (2003). Reliability and validity of the Dutch version of QUEST 2.0 with users of various types of assistive devices. Disabil Rehabil.

[18] Demers L, Monette M, Lapierre Y, Arnold DL, Wolfson C (2002). Reliability, validity, and applicability of the Quebec User Evaluation of Satisfaction with assistive Technology (QUEST 2.0) for adults with multiple sclerosis. Disabil Rehabil.

[19] Gauthier- Gagon C, Grise MC (2001). Tools for outcome measurement in lower limb amputee rehabilitation.

[20] Heinemann AW, Bode RK, O'Reilly C (2003). Development and measurement properties of the Orthotics and Prosthetics Users' Survey (OPUS): a comprehensive set of clinical outcome instruments. Prosthet Orthot Int..

[21] World Health Organization. Process of translation and adaptation of instruments 2007. http://www.who.int/substance_abuse/research_tools/translation/en/ Accessed 12 Jan 2016.

[22] Brace N, Kemp R, Snelgar R (2009). SPSS for psychologists, 4th edition edn.

[23] Wallin A-M, Ahlström G (2006). Cross-cultural interview studies using interpreters: systematic literature review. J Adv Nurs.

[24] Krippendorff K (2004). Content Analysis as Introduction to Its Methodology.

[25] Jensen JS, Nilsen R, Zeffer J (2005). Quality benchmark for trans-tibial prostheses in low-income countries. Prosthet Orthot Int.

[26] Matsen SL (1999). A closer look at amputees in Vietnamn: a field survey of Vietnamese using prosthesis. Prosthet Orthot Int.

[27] Brakel WH, Poetsma PA, Tam PT, Verhoeff T (2010). User satisfaction and use of prostheses in ICRC's special fund for the disabled project in Vietnamn. Asia Pacific Disability Rehabilitation Journal.

[28] Eide AH (2013). Users of the physical rehabilitation services supported by ICRC Special Fund for the Disabled in Vietnam. Description and assesments of impact and future service needs.

[29] Peethambaran A (2000). The relationship between performance, satisfaction, and well being for patients using anterior and posterior design knee-ankle-foot-orthosis. J Prosthet Orthot.

[30] Radtka SA, Oliveira GB, Lindstrom KE, Borders MD (2006). The kinematic and kinetic effects of solid, hinged, and no ankle–foot orthoses on stair locomotion in healthy adults. Gait Posture.

[31] Alimusaj M, Fradet L, Braatz F, Gerner HJ, Wolf SI (2009). Kinematics and kinetics with an adaptive ankle foot system during stair ambulation of transtibial amputees. Gait Posture.

[32] Su P-F, Gard SA, Lipschutz RD, Kuiken TA (2010). The Effects of Increased Prosthetic Ankle Motions on the Gait of Persons with Bilateral Transtibial Amputations. Am J Phys Med Rehabil.

[33] Sin SW, Cowe DHK, Cheng JCY (2001). Significance of non -level walking on transtibial prosthesis fitting with particular reference to the effects of anterior-posterior alignment. J Rehabil Res Dev.

[34] Sjödahl C, Jarnlo G-B, Persson BM (2001). Gait improvment in unilateral transfemoral amputees by a combined psychological and physiotherapeutic treatment. J Rehabil Med.

[35] Campbell DJT, Coll N, Thurston WE (2012). Considerations for the provision of prosthetic services in post-disaster contexts: the Haiti Amputee Coalition. Disabil Soc.

[36] International Society for Prosthetics and Orthotics (2001). Report of ISPO Consensus Conference on Appropriate Orthopaedic Technology for Low-Income Countries Moshi, Tanzania, 18-22 September, 2000. Prosthet Orthot Int.

[37] Jensen JS, Sexton S (2010). Appropriate Prosthetic and Orthotic Technologies. In.

[38] Ghoseiri K, Bahramian H (2011). User satisfaction with orthotic and prosthetic devices and services of a single clinic. Disabil Rehabil.

[39] Geertzen JHB, Gankema HGJ, Groothoff JW, Dijkstra PU (2002). Consumer satisfaction in prosthetics and orthotics facilities. Prosthet Orthot Int.

[40] Bosmans J, Geertzen J, Dijkstra PU (2009). Consumer satisfaction with the services of prosthetics and orthotics facilities. Prosthet Orthot Int.

[41] Pezzin L, Dillingham TR, MacKenzie EJ, Ephraim P, Rossbach P (2004). Use and satisfaction with prosthetic limb devices and related services. Arch Phys Med Rehabil..

[42] Karmarkar AM, Collins DM, Wichman T, Franklin A, Fitzgerald SG, Dicianno BE, Pasquina PF, Cooper RA (2009). Prosthesis and wheelchair use in veterans with lower-limb amputation. J Rehabil Res Dev.

[43] Berke GM, Fergason J, Milani JR, Hattingh J, McDowell M, Nguyen V, Reiber GE (2010). Comparison of satisfaction with current prosthetic care in veterans and servicemembers from Vietnam and OIF/OEF conflicts with major traumatic limb loss. J Rehabil Res Dev.

[44] Landmine Survivors Network (2006). Prosthetics and orthotics program guide implementing P&O services in low-income settings. A guide for planners and providers of services for persons in need of orthopeadic devices.

[45] World Health Organization, United States Agency for International Development (2011). Joint position paper on the provision of mobility devices in less resourced settings: a step towards implementation of the convention on the rights of persons with disabilities (CRPD) related to personal mobility.

[46] Global petrol prices. Diesel prices 2016. http://www.globalpetrolprices.com/diesel_prices/. Accessed 12 Jan 2016.

[47] Van Rooy G, Amadhila EM, Mufune P, Swartz L, Mannan H, MacLachlan M (2012). Perceived barriers to accessing health services among people with disabilities in rural northern Namibia. Disabil Soc.

[48] Schneider M, Eide AH, Amin M, MacLachlan M, Mannan H. Inclusion of vulnerable groups in health policies: Regional policies on health priorities in Africa. African Journal of Disability. 2013; 2(1):9. http://dx.doi.org/10.4102/ajod.v2i1.40. 10.4102/ajod.v2i1.40 PMC5442580 28729986

[49] Pascore CG (1983). Patient satisfaction in primary health care: A literature review and analysis. Eval Program Plann.

[50] Kark L, Simmons A (2011). Patients satisfaction following lower-limb amputation: the role of gait deviation. Prosthet Orthot Int.

[51] Keith RA (1998). Patient satisfaction and rehabilitation services. Arch Phys Med Rehabil.

[52] Andregård E, Magnusson L. Experiences of attitudes in Sierra Leone from the perspective of people with poliomyelitis and amputations using orthotics and prosthetics. Disabil Rehabil. 2016;1–7. 10.1080/09638288.2016.1236409 27829289

[53] Sierra Leone Ethics and Scientific Review Committee: Sierra Leone Ethics and Scientific Review Committee-Guidelines and Checklist. In*.* Freetown: Connaught Hospital; 2011.

[54] National Research Counsil of Malawi. National Health Sciences Research Committee Malawi. Procedures and guidelines for the conduct of research in Malawi Lilongwe: National Research Counsil of Malawi; 2003. http://www.sdnp.org.mw/nrcm/policies/guideline3.htm. Accessed 12 Jan 2016.

