# Supplementary material for: Patients’ Satisfaction with Lower-limb Prosthetic and Orthotic Devices and Service delivery in Sierra Leone and Malawi
Source: BMC Health Serv Res. 2017 Feb 1;17:102. doi: 10.1186/s12913-017-2044-3 (PMC5286686; doi:10.1186/s12913-017-2044-3)
Supplement: Additional file 1: — Supplementary file the Questionnaire Prosthetic and Orthotic Device and Services. The questionnaire includes questions regarding the characteristics of participants and assistive devices, mobility, pain and satisfaction with assistive device. (DOCX 19 kb) [file 12913_2017_2044_MOESM1_ESM.docx]

**Prosthetic and Orthotic Device and Services**

| B1 Country | Name of Beneficiary | | |
| --- | --- | --- | --- |
| B2 Centre | B3 Age | B4 Gender □ F □M | |
| B5 Date of evaluation | B6 Home Address: □ City □ Village/Rural area | | |
| B7 Name of evaluator |  |  |  |
| B8 Translator □ Yes □No | B9 Ethnic group | | B10 Religion |
| B11 Prostetist orthoptist female or male □ F □M | B12 Income □ No income □ Sometimes income □ Regular income from employment | | |
| B13Year of Amputation / disability: | B14 Type of disability: | | |

Code number:

Type of prosthesis/ orthoses:

| B15 Left  □ TT □ KD /TF □ AFO □ KAFO | B16 Do you have a spare device available?  □Yes □No |
| --- | --- |
| B17 Right  □ TT □ KD /TF □ AFO □ KAFO | B17 Do you have a spare device available?  □Yes □No |

| At present general condition of device?  B19 Left  □ Never used □ Broken cannot be used □ In use but needs repair □ In use good condition  B20 Right  □ Never used □ Broken cannot be used □ In use but needs repair □ In use good condition |
| --- |

| How many hours a day do you use your device / spare device?  B21 Left  B22 Right |
| --- |

| B23Do you use crutches? □ Yes instead of device □ Yes together with device □ No |
| --- |
| B24Do you use a wheelchair? □ Yes instead of device □ Yes together with device □ No |

| B25 How far can you walk without your assistive device?  □Not at all □A few meters □About 100 meters □A longer distance about a kilometer or more |
| --- |
| B26 How far can you walk with your assistive device?  □Not at all □A few meters □About 100 meters □A longer distance about a kilometer or more |

| B27 I have the ability to pay for costs associated with receiving the service (appliances, accommodation travel other)? □Yes □No |
| --- |

For each of the items please rate your satisfaction using the scale of 1 to 5.

| **1** | **2** | **3** | **4** | **5** |
| --- | --- | --- | --- | --- |
| **Not satisfied at all** | **Not very satisfied** | **More or less satisfied** | **Quite satisfied** | **Very satisfied** |

| v1. How satisfied are you with the training you received with your assistive device?  Comments: | 1 2 3 4 5 |
| --- | --- |
| v2. How satisfied are you with the coordination of P&O services with other rehabilitation professionals (Physiotherapist CBR worker, Doctor others)?  Comments: | 1 2 3 4 5 |
| v3. How satisfied are you with, the looks / cosmesis of your assistive device?  Comments: | 1 2 3 4 5 |
| v4. How satisfied are you with how easy it is to keep your assistive device clean?  Comments: | 1 2 3 4 5 |

For each of the items X alternative that is most true for you.

| v5. My assistive device causes me pain while using it?  □ Always □Often □ Seldom □ Never □ Not applicable  Comments: |
| --- |
| v6. My assistive device causes me wounds or skin irritations?  □ Always □ Often □ Seldom □ Never □ Not applicable  Comments: |

For each of the items please X the alternative that is most true for you.

| v7. I have the ability to rise from a chair?  □ Yes, without any difficulty □ Yes, with difficulty □ No, not at all □ Not applicable  Comments: |
| --- |
| v8. I have the ability to move around in my home?  □ Yes, without any difficulty □ Yes, with difficulty □ No, not at all □ Not applicable  Comments: |
| v9. I have the ability to walk on uneven ground/roads?  □ Yes, without any difficulty □ Yes, with difficulty □ No, not at all □ Not applicable  Comments: |
| v10. I have the ability to walk up and down a hill?  □ Yes, without any difficulty □ Yes, with difficulty □ No, not at all □ Not applicable  Comments: |
| v11. I have the ability to walk on stairs?  □ Yes, without any difficulty □ Yes, with difficulty □ No, not at all □ Not applicable  Comments: |
| v12. I have the ability to get in and out of a car?  □ Yes, without any difficulty □ Yes, with difficulty □ No, not at all □ Not applicable  Comments: |
| v13. I have the ability to get in and out of a bus?  □ Yes, without any difficulty □ Yes, with difficulty □ No, not at all □ Not applicable  Comments: |

| v14. I have the possibility to access the workshop (distance, transport costs or availability, lack of assistance other barriers)?  □ Completely true □ Sometimes true □ Completely false □ Not applicable  Comments: |
| --- |
| v15. The prosthetist orthotist or technician gives me the opportunity to express my views about my assistive device (prosthesis/ orthosis)?  □ Completely true □ Sometimes true □ Completely false □ Not applicable  Comments: |
| v16. I trust and have confidence that my prosthetist orthotist is capable of delivering a quality service?  □ Completely true □ Sometimes true □ Completely false □ Not applicable  Comments: |

© Lina Magnusson 2013, for permission to use please contact [Lina.Magnusson@med.lu.se](mailto:Lina.Magnusson@med.lu.se) [magnussonlina@hotmail.com](mailto:magnussonlina@hotmail.com)
